# Modelling epidemic spread in cities using public transportation as a proxy for generalized mobility trends

**DOI:** 10.1038/s41598-022-10234-8

**Published:** 2022-04-16

**Authors:** Omar Malik, Bowen Gong, Alaa Moussawi, Gyorgy Korniss, Boleslaw K. Szymanski

**Affiliations:** 1 Department of Physics, Applied Physics, and Astronomy, Rensselaer Polytechnic Institute, Troy, NY 12180 USA; 2 Network Science and Technology Center, Rensselaer Polytechnic Institute, Troy, NY 12180 USA; 3 New York City Council, City Hall Park, New York, NY 10007 USA; 4 Department of Computer Science, Rensselaer Polytechnic Institute, Troy, NY 12180 USA

**Keywords:** Statistical physics, Computational models, Epidemiology

## Abstract

We study how public transportation data can inform the modeling of the spread of infectious diseases based on SIR dynamics. We present a model where public transportation data is used as an indicator of broader mobility patterns within a city, including the use of private transportation, walking etc. The mobility parameter derived from this data is used to model the infection rate. As a test case, we study the impact of the usage of the New York City subway on the spread of COVID-19 within the city during 2020. We show that utilizing subway transport data as an indicator of the general mobility trends within the city, and therefore as an indicator of the effective infection rate, improves the quality of forecasting COVID-19 spread in New York City. Our model predicts the two peaks in the spread of COVID-19 cases in NYC in 2020, unlike a standard SIR model that misses the second peak entirely.

## Introduction

Long-range mobility, such as traveling between cities, can cause a disease to spread through case importation across large distances^1, 2^. Short-range mobility, such as usage of city buses or trams, has been correlated with a higher risk of contracting acute respiratory infections^3^ and with the number of cases of COVID-19 within cities^4, 5^. Accordingly, restrictions on human mobility, either directly by shutting down public transportation^6, 7^ or indirectly by restricting public and private gatherings^8^, which were highly effective in stopping the spread of COVID-19. We hypothesize that alongside being a high-risk medium for infections, public transportation usage is also a good indicator for the level of short-range mobility for the entire population of a city.

### COVID-19 in New York City

When it became clear that the COVID-19 virus is highly infectious, New York City (NYC) imposed restrictions that included shutting-down non-essential businesses and forbidding large gatherings, but kept public transportation^9^ and schools open^10^. The usage of NYC’s sprawling subway system was found to be correlated with the spread of COVID-19^4, 5, 11^ and mobility patterns in general were correlated with the spread of COVID-19 within regions of the city^12, 13^. There are various models of disease spread that incorporate human mobility patterns, such as a recent disease transmission model inspired by collision theory gas-phase chemistry^14^, or a metapopulation model that allows for the movement of individuals between subpopulations^15^. We propose a model based on SIR dynamics where we explicitly model human mobility as a parameter and treat the infection rate as a function of the mobility of a region. To effectively model the spread of COVID-19 in NYC, we focus on data from the NYC subway. We hypothesize that trends in subway usage are correlated with the usage of other modes of transport, such as buses, taxis etc. We therefore treat subway usage as an indicator for broader human mobility patterns in the city.

## Data

### New York City subway turnstile data

Unlike pedestrian traffic, private automobiles, and to some extent taxis, public transportation, and in particular the subway, has detailed records of passenger traffic such as the total number of entries and exits from a station collected in real time. This enables us to extract some important statistics regarding passenger traffic using publicly available data on subway usage published by the Metropolitan Transportation Authority (MTA)^16^. As awareness of the pandemic grew in early 2020 the Governor of New York announced a state of emergency on March 7 2020, followed shortly by the passage of an executive order, known as ’New York State on PAUSE’, that shutdown all non-essential businesses in the state^9, 17^. New York City saw a decline in subway usage alongside various other modes of public transport, including bikeshares^18, 19^, and taxis^20^. As restrictions were slowly eased in the later half of the year, there was a corresponding increase in the usage of public transport, although some modes were preferred over others at different rates than before the pandemic. Bikehares, for example, had recovered to their 2019 levels by September 30, 2020 while subway ridership was at 30$\%$ of pre-pandemic levels^18^. Despite the different rates at which different modes recovered, both bikeshares and the subway saw increases in usage in the latter half of 2020. We believe that this increase in both modes of transport corresponds to the underlying increase in human mobility as restrictions were loosened after June 8, 2020. This motivates us to use this directly measurable traffic as a proxy for all traffic in the city.

We collected and analyzed the subway turnstile data of New York City for 12 consecutive months, starting from January 2020 to December 2020. The MTA publishes turnstile data on a weekly basis, which includes administrative information such as the control area, unit number, station name and line name, as well as the counts of the entries and exits at a specific time for a particular turnstile^16^. The system collects these counts every four hours, each of which is a cumulative register value. The data were first converted into dataframes and then into a combination of control area code, remote unit, subunit channel position (SCP), as well as the time of the observation that serves as a unique ID to identify and remove duplicate records. We removed entries related to Port Authority Trans-Hudson (PATH) trains, since they do not represent the mobility among NYC boroughs. The absolute difference between the first and last counts at a turnstile on a particular day defines the number of subway riders passing through that particular turnstile. The geographic coordinates of the station and the borders of each borough allow us to place each station in its corresponding borough. We calculated the total number of borough-level subway riders by summing the numbers of riders at all the turnstiles of all subway stations located within each borough. To estimate the mobility between boroughs, we used a survey that was conducted among subway riders regarding the origin and destination of their trips^21^. Given the number of departures at a given station, we used probabilities extracted from the survey to determine the destinations of those trips.

### COVID-19 data

**Figure 1:**
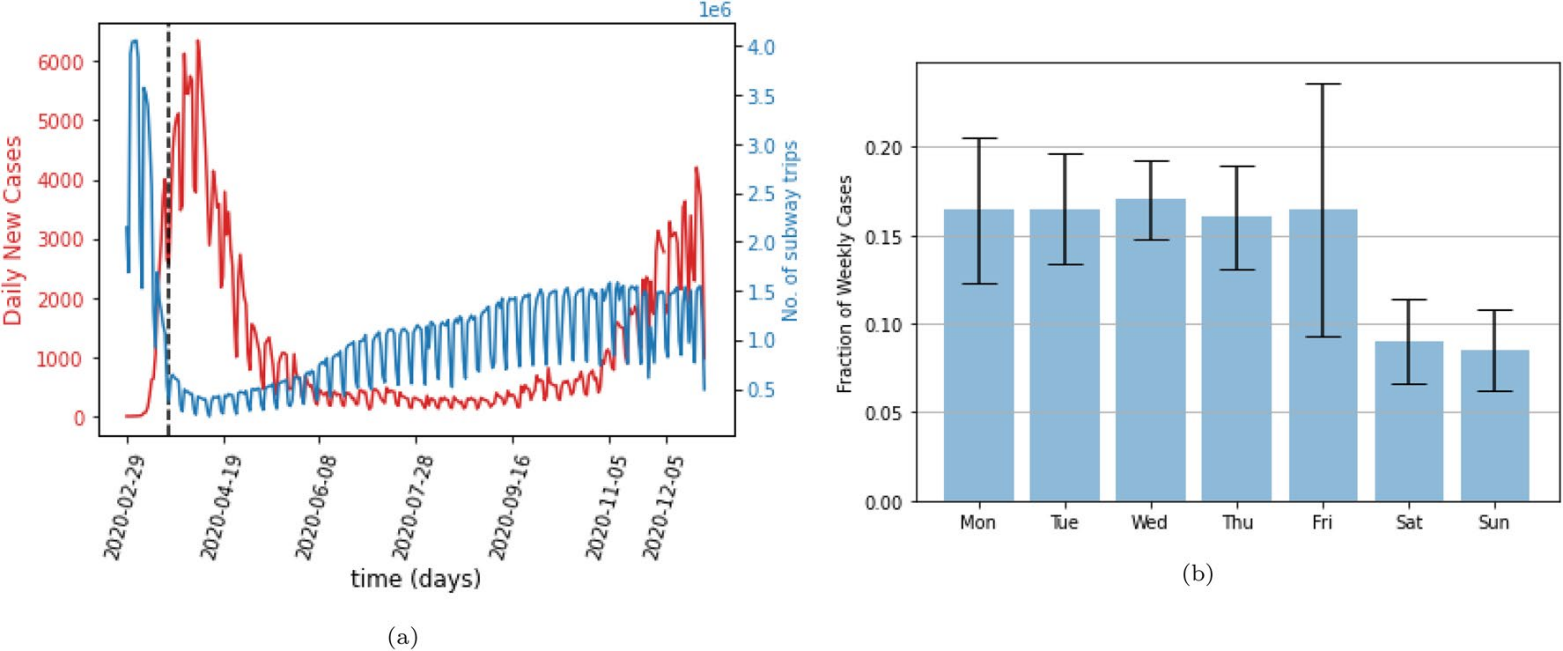
(**a**) The red line shows the daily reported cases of COVID-19 in New York City. The blue line shows the total daily number of trips taken on the subway, with entries related to the Port Authority Trans-Hudson (PATH) removed. The dotted line indicates the start of the NY PAUSE Program, (**b**) The fraction of total weekly cases reported on each day of the week, averaged over 44 weeks. While the weekdays remain largely consistent, there is a significant drop in reporting on weekends.

We used publicly available data published by the NYC government about the number of new COVID-19 cases reported for each day and for each of the five boroughs^22^. Figure 1a shows that this data has a clear weekly cyclical pattern. Figure 1b shows that this pattern arises because of the much smaller numbers of cases that were reported on weekends than on weekdays. We remove this pattern by using a running 7-day average of the number of daily cases.

We also chose to restrict our analysis to 2020, since the introduction of vaccines in early 2021 decreases the number of people susceptible to infection. To account for this decrease on the spread of epidemics would require the introduction of another parameter that might change the quantitative effect of mobility on the spread of the disease.

### Population data

All population data for New York City were taken from the 2020 census conducted by the US Census Bureau and published on their website^23^.

## Model

We start with the well-established SIR model^24, 25^. While more powerful models for modelling disease spread exist, such as the SEIR model^26^, we picked the SIR model in order to reduce the number of parameters and avoid overfitting. The COVID-19 hospitalization data that we use only reports daily newly infected cases and we do not believe this data is fine-grained enough to justify the use of a more complex model. The SIR model divides the total population (*N*) into susceptible (*S*), infected (*I*), and recovered or dead (*R*) compartments. The equations governing the spread of the disease are

1 $$\begin{aligned} \dfrac{d}{dt}S(t)&= -\beta \dfrac{S(t) I (t)}{N}, \end{aligned}$$

2 $$\begin{aligned} \dfrac{d}{dt}I(t)&= \beta \dfrac{S(t) I (t)}{N} - \gamma I(t), \end{aligned}$$

3 $$\begin{aligned} \dfrac{d}{dt}R(t)&= \gamma I(t), \end{aligned}$$

where $\beta$ and $\gamma$ are the infection and recovery rate, respectively.

We modify the model by dividing the total population into subpopulations called regions, each with a fraction of the population $p_j$ living in region *j*, thus $\sum _j p_j = 1$. When we apply the model to New York City the different $p_j$ represent the populations of the five boroughs of New York City, normalized by the total population of the city, which was 8,804,190 in 2020^23^. Within a region, people will have different infection rates based on their activity. The infection rate for individuals working from home and following strict quarantine protocols will be lower than the rate for frontline workers. Each activity-based cohort has an associated infection rate $\beta _{jc}$. Additionally, people may also have access to different quality of healthcare, which may impact the frequency of testing and the likelihood of visiting a doctor. Both of these parameters influence the recovery rate of a patient. Each healthcare-based cohort has an associated recovery rate $\gamma _{je}$. The fraction of the total population in region *j* with behavior *c* and healthcare *e* is denoted as $p_{jce}$. For parameters representing the population fractions, an omitted index indicates a sum over all values of this index, so $p_{jc} = \sum _{e} p_{jce}$ and $p_j = \sum _{c, e} p_{jce}$ etc.

Each cohort within each region follows SIR dynamics. The equations governing the population fractions of susceptible, infected and recovered individuals are given by:

4 $$\begin{aligned} s_{jce}(t + \Delta t)&= s_{jce}(t)\left( 1 - \beta _{jc}i_{jce}(t) \Delta t \right) , \end{aligned}$$

5 $$\begin{aligned} i_{jce}(t + \Delta t)&= i_{jce}(t) + \left( \beta _{jc}s_{jce}(t)i_{jce}(t) - i_{jce}(t)\gamma _{je} \right) \Delta t, \end{aligned}$$

6 $$\begin{aligned} r_{jce}(t + \Delta t)&= r_{jce}(t) + i_{jce}(t)\gamma _{je} \Delta t, \end{aligned}$$

where $s_{jce}(t), i_{jce}(t)$ and $r_{jce}(t)$ are the population fractions of susceptible, infected and recovered individuals, respectively at time *t*.

### Inter-region mixing

So far our model follows straightforward SIR dynamics. We now want to introduce inter-region mixing through a mixing parameter that tells us the population fraction of one region that is visiting another region at a given time. In order to calculate this quantity, we need to know the origin, destination, and trip duration for every rider using the subway. From the data, we only know the borough of departure. We do not know an individual rider’s destination based just on the borough they departed from. Using an MTA survey on the use of the NYC subway, we can determine the probabilities of a trip departing and terminating at different boroughs^21^.

In order to determine the average time spent visiting a borough, we also need to know the borough of origin of riders arriving at a station. From the survey, we know $P(o_j)$, the probability of any trip originating in borough *j*, $P(d_j)$, the probability of any trip terminating in borough *j*, and $P(d_{j'} | o_j)$, the probability that a trip originating in borough *j* terminates in borough $j'$^21^.

It will be helpful to define a fractional time, $\tau$, which measures the time of day as the fraction of the day that has passed since midnight. So, for example, 3 PM corresponds to a fractional time of $\tau = 0.625$. In order to determine the average duration that residents of one borough spend in another borough, we start by treating NYC as a closed system where individuals do not travel into or out of the city, and all residents of a borough return to it at the end of the day. If a rider *k* leaves borough *j* at fractional time $\tau _{A_k}$ and returns at $\tau _{D_k}$, then the fraction of the day spent away from the borough is $\tau _{A_k} - \tau _{D_k}$.

If there are $M_t$ total riders on day *t*, then the average fraction of the day spent away from the borough on that day is $\dfrac{\sum _{k=1}^{M_t}(\tau _{A_k} - \tau _{D_k})}{M_t}$. This average can be rewritten by collecting all the arrival and departure times separately rather than tracking each rider’s individual arrival and departures so that $\dfrac{\sum _{k=1}^{M_t}\tau _{A_k} - \sum _{k=1}^{M_t} \tau _{D_k}}{M_t}$. The subway turnstile data does not track the arrival and departure of individual riders. Instead, it provides a number of snapshots everyday of the cumulative arrivals and departures. So, the data instead provides us with the number of arrivals, $A_{t, j}(\tau _k)$, and departures, $D_{t, j}(\tau _k)$, at fractional time $\tau _k$, where the index *k* no longer refers to riders, but to the different times at which the number of entries and exits are recorded. We can then write the average fractional time spent by residents of borough *j* away from their home borough

7 $$\begin{aligned} {\Delta \tau _{j} =\dfrac{1}{t_{{\rm tot}}}\sum _{t=1}^{t_{{\rm tot}}} \dfrac{\sum _{k}\tau _k\left( A_{t, j}(\tau _k) - D_{t, j}(\tau _k)\right) }{ M_t},} \end{aligned}$$

where $t_{{\rm tot}}$ is the total number of days. It should be noted that the sum over *k* is no longer over the number of riders, but over the number of snapshots of total entries and exits taken that day.

For a variety of reasons, such as travel into and out of the city and the usage of multiple modes of transport, the number of arrivals and departures at a station will not match exactly. In order to account for this, we match the number of arrivals and departures at a given snapshot in time in the data, and any discrepancy is added to the next time bin. Once all time periods have been accounted for, any unmatched arrivals or departures are ignored. The equation then becomes

8 $$\begin{aligned} {\Delta \tau _{j} = \dfrac{1}{t_{{\rm tot}}}\sum _{t=1}^{t_{{\rm tot}}}\dfrac{\sum _{k}\tau _k\text {min}\left( \tilde{A}_{t, j}(\tau _k), \tilde{D}_{t, j}(\tau _k)\right) }{ \tilde{M}_t}}, \end{aligned}$$

where

9 $$\begin{aligned} \tilde{A}_{t, j}(\tau _k)&= A_{t, j}(\tau _k) + {U^A_t(\tau _k)}, \end{aligned}$$

10 $$\begin{aligned} \tilde{D}_{t, j}(\tau _k)&= D_{t, j}(\tau _k) + {U^D_t(\tau _k)}, \end{aligned}$$

11 $$\begin{aligned} {U^D_t(\tau _k)}&= {\text {max}\left( 0, \tilde{D}_{t, j}(\tau _{k - 1}) - \tilde{A}_{t, j}(\tau _{k - 1}) \right) }, \end{aligned}$$

12 $$\begin{aligned} {U^A_t(\tau _k)}&= {\text {max}\left( 0, \tilde{A}_{t, j}(\tau _{k - 1}) - \tilde{D}_{t, j}(\tau _{k - 1}) \right) }, \end{aligned}$$

13 $$\begin{aligned} {U^D_t(\tau _0)}&= {U^A_t(\tau _0)} = 0, \end{aligned}$$

14 $$\begin{aligned} \tilde{M}_t&= {\text {min}\left( \sum _{k} A_{t, j}(\tau _k), \sum _{k} D_{t, j}(\tau _k)\right) }, \end{aligned}$$

where $U^A_t(\tau _k)$ and $U^D_t(\tau _k)$ are the unmatched arrivals and departures from the previous time period. An example of the matching process is shown in Table 1. We can now write our mixing parameter

15 $$\begin{aligned} f_{jj'}(t)&= {\Delta \tau _{j} P(d_{j'} | o_{j}) \sum _{k} \dfrac{D_{t, j}(\tau _k)}{N}}. \end{aligned}$$

On any given day we could estimate the number of people, expressed as a fraction of the total population, that travel from borough *j* to borough $j'$ by $P(d_{j'} | o_j)\sum _{k}\dfrac{D_{t, j}(\tau _k)}{N}$. This would give us an estimate of how many of the people leaving borough *j* are heading towards $j'$. However, we do not have detailed temporal resolution on the movement of riders within a borough and we do not know when any individual rider returns to their home borough. We define an effective population of visitors by multiplying this quantity with $\Delta \tau _{j}$, the estimate of the average time fraction spent away from borough *j*, that spend the entire day in borough $j'$. The mixing parameter represents this effective visiting population.

The population fraction that leaves region *j* for all other regions is $f_j^- = \sum _{j' \ne j} f_{jj'}$, while the population fraction that arrives at region *j* from all other regions is $f_j^+ = \sum _{j' \ne j}f_{j'j}$. The resulting total population fraction in region *j* becomes $p_j + f_j^+ - f_j^-$.

**Table 1:** An example of the matching process using real data. The columns labelled ENTRIES, EXITS, and TIME are from the turnstile data. We calculate the number of arrivals, $A_{t, j}(\tau )$, by subtracting successive values of the running total of entries. These arrivals are assigned a fractional time, $\tau$, corresponding to the midpoint of successive time snapshots. The departures, $D_{t, j}(\tau )$, are calculated in the same way.

Entries	Exits	Time	$\tau$	$A_{t, j}(\tau )$	$D_{t, j}(\tau )$	$U^A_t(\tau )$	$U^D_t(\tau )$	$\tilde{A}_{t,j}(\tau )$	$\tilde{A}_{t,j}(\tau )$
0007328037	0002483731	03:00:00							
0007328044	0002483742	07:00:00	0.208	7	11	0	0	7	11
0007328075	0002483781	11:00:00	0.375	31	39	0	4	31	43
0007328193	0002483821	15:00:00	0.542	118	40	0	11	118	51
0007328375	0002483878	19:00:00	0.708	182	57	67	0	249	57
0007328499	0002483910	23:00:00	0.875	124	32	192	0	316	32

We must now keep track of the part of the susceptible and infected populations of region *j* that do not leave the region, which we call ’stationary’, given by

16 $$\begin{aligned} s_{jce}^S = s_{jce}\dfrac{p_j - f_j^-}{p_j}, \end{aligned}$$

17 $$\begin{aligned} i_{jce}^S = i_{jce}\dfrac{p_j - f_j^-}{p_j}. \end{aligned}$$

It should be noted that while this ’stationary’ population does not leave the borough, the individuals that constitute this population may still be mobile within their borough. This will be addressed later in this section. We also keep track of infected individuals visiting region *j* from other regions. These are given by

18 $$\begin{aligned} i_{jce}^+&= \sum _{j' \ne j} i_{j'ce} \dfrac{f_{j'j}}{p_{j'}}. \end{aligned}$$

We can now write down the equations for the stationary susceptible and infected populations for region *j*:

19 $$\begin{aligned} \begin{aligned} s_{jce}^S(t + \Delta t) = s_{jce}^S(t)(1 - \beta _{jc}i_{jce}^S(t)\Delta t - \beta _{jc}i_{jce}^+(t)\Delta t ). \end{aligned} \end{aligned}$$

We also need to track individuals from region *j* who are visiting region $j'$. These are given by

20 $$\begin{aligned} s_{jce}^{V} = \sum _{j' \ne j} s_{jce}^{j'} = \sum _{j' \ne j} s_{jce}\dfrac{f_{jj'}}{p_j}. \end{aligned}$$

These individuals will interact with stationary infected individuals from other regions. We can now write the equations for the individuals from region *j* visiting all other regions

21 $$\begin{aligned} \begin{aligned} s_{jce}^V(t + \Delta t) = \sum _{j' \ne j}s_{jce}^{j'}(t)(1 - \beta _{j'c}i_{j'ce}^{+}(t)\Delta t - \beta _{j'c}i_{j'ce}^S(t)\Delta t), \end{aligned} \end{aligned}$$

We can combine the equations for the stationary and visiting populations by introducing a flow parameter

22 $$\begin{aligned} \begin{aligned} \lambda _{jce} =&s_j^{S}\beta _{jc}\left[ i_{jce}^S + i_{jce}^+ \right] + \sum _{j' \ne j}s_j^{j'} \beta _{j'c}\left[ i_{j'ce}^{+} + i_{j'ce}^S \right] . \end{aligned} \end{aligned}$$

The flow parameter lets us compactly write the dynamics of region *j*

23 $$\begin{aligned} \begin{aligned} s_{jce}(t + \Delta t) =&s_{jce}(t) - \lambda _{jce}(t)\Delta t, \end{aligned} \end{aligned}$$

24 $$\begin{aligned} \begin{aligned} i_{jce}(t + \Delta t) =&i_{jce}(t) + \left( \lambda _{jce}(t) - i_{jce}(t)\gamma _{je} \right) \Delta t, \end{aligned} \end{aligned}$$

25 $$\begin{aligned} \begin{aligned} r_{jce}(t + \Delta t) =&r_{jce}(t) + i_{jce}(t)\gamma _{je}\Delta t. \end{aligned} \end{aligned}$$

**Figure 2:**
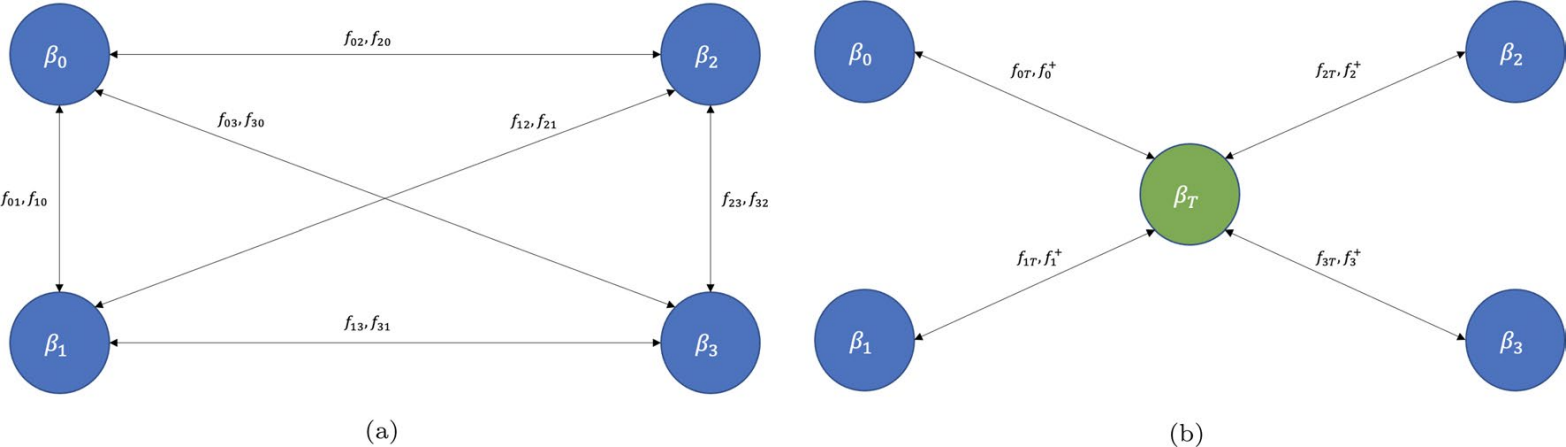
(**a**) Schematic representation of the mobility-based SIR model. Each region *j* has an associated infection rate $\beta _j$ and mobility parameters $f_{jj'}$ and $f_{j'j}$, which represent individuals from region *j* visiting region $j'$ and vice versa. (**b**) The enhanced model that includes a public transportation node without a permanent population. Inter-region mixing still occurs as in the basic model, but the visiting populations of every region pass through the transportation node for the duration of their commute time during which they are exposed to the higher infection rate associated with using public transportation. The effective population of region *j* that is commuting is given by $f_{jT}$ while the effective population of all other regions that are visiting region *j* are given by $f_{j}^+$.

Since the data provided by the NY government tracks the number of newly reported cases and does not report the number of active cases (*i*(*t*) in our model) we construct the quantity

26 $$\begin{aligned} i_{jce}^{{\rm new}}(t)&= \sum _{t' = t}^{t + 1-\Delta t}\lambda _{jce}(t')\Delta t, \end{aligned}$$

where *t* is in units of days. In other words, $i_{jce}^{{\rm new}}(t)$ represents the number of new cases reported on day *t* and this is the quantity that we will fit to the data. Figure 2a shows a schematic representation of our mobility-based SIR model.

### Introducing a public transportation node

While our model accounts for the spread of disease through the transit of infected individuals between regions, it does not take into account that use of public transportation poses a higher risk of infection^3^. To account for this effect, we introduce a public transportation node, denoted by the index *T*. The fraction of the population permanently residing on this node is 0 ($p_T = 0$). We modify our model so that all riders travelling to another borough spend some part of their time at node *T*. This duration is taken from the average commute time reported by riders of each borough^21^. The mixing parameter from node *j* to node *T* becomes

27 $$\begin{aligned} {f_{jT}(t) = \Delta \tau _{jT}\sum _{j' \ne j} P(d_{j'} | o_j) \sum _{k} \dfrac{D_{t, j}(\tau _k)}{N}}, \end{aligned}$$

where $\Delta \tau _{jT}$ is the commute time for riders in node *t*, expressed as a fraction of the day. Due to the introduction of a transport node we must also modify our expression for the inter-borough mixing parameter, which becomes

28 $$\begin{aligned} {f_{jj'}(t) = \left( \Delta \tau _{j} - \Delta \tau _{jT}\right) P(d_{j'} | o_j) \sum _{k} \dfrac{D_{t, j}(\tau _k)}{N}}. \end{aligned}$$

Figure 2b shows a schematic depiction of the model with the public transportation node. The introduction of such a node allows us to independently model the infection rate during rides on public transportation systems, $\beta _T$, for individuals using public transportation. Our model does not track individual interactions, but only the infection rate at the population level. We introduce the transport node to model the different rates of infection experienced by the fraction of the population that uses the subway, where they interact with a different mixture of populations than the mixture that they encounter in the boroughs in which they live and work.

### Mobility-dependent infection rate

While inter-region mixing and the introduction of a public transportation node account for mobility between regions, we also need to account for mobility within a region. To do this, we introduce a mobility parameter for each region, $m_j(t)$, which represents the extent to which individuals are moving within the region. We then write our infection rate as

$$\begin{aligned} \beta _{jce}(t) = \beta ^0_{jce}m_j(t), \end{aligned}$$

where $\beta ^0_{jce}$ is the static infection rate. For the particular case of the NYC subway, $m_j(t)$ is calculated by taking a 7-day moving average of the total trips that start in borough *j* and rescaling this quantity by dividing it by the maximum number of trips taken in one day in borough *j* in this training period, thereby scaling it between 0 and 1. A plot of the average mobility parameter, defined as $m_{{\rm avg}}(t) = \sum _j p_j m_j(t)$, is plotted in Fig. 4b. We are using the level of subway usage as a stand-in for all short-range mobility. We found that the number of bike-share rides taken during the pandemic was correlated with the number of subway trips^19^. We assume that subway usage is correlated with all mobility within the city, even as subway usage fell during the pandemic across cities around the world^27^.

## Results

While our model is able to incorporate complex demographic information such as healthcare status, access to testing, and public policies regarding gathering sizes and mask usage, we are limited by the data to which we have access. Since we only have public transportation data and the daily case count, we will assume that each region in our model, corresponding to one of the five boroughs of NYC, has a uniform demographic distribution. This means that we will be ignoring the *c* and *e* indices in our model.

In order to model the effect of different policies, we pick March 22, 2020, the official start day of the NY PAUSE Program, as the beginning of the lockdown. We assume that there are *two different* infection rates, one before and the other after this date. This assumption is made because the PAUSE program marks the start of the implementation of widespread mask usage and social distancing. These are non-mobility factors which impact the overall infection rate.

We also assume that the intensity of usage of public transportation is correlated with the infection rate. The infection rate for borough *j* then becomes

29 $$\begin{aligned} \beta _j(t) = \beta ^{{\rm p}}(t) {m_{j}(t- {t_D})}, \end{aligned}$$

where $\beta ^{{\rm p}}(t) = \beta ^{{\rm h}}$ before the start of the NY PAUSE Program on March 22 2020, and $\beta ^{{\rm p}}(t) = \beta ^{{\rm l}}$ afterwards. The second term, ${m_{j}(t- {t_D})}$, represents the normalized daily number of trips taken on the subway within a region. The parameter ${t_D}$ accounts for the population level delay between subway usage and the subsequent increase in Covid-19 cases. We also average $\beta _j(t)$ over a 7 day moving window in order to smooth out abrupt changes due to both noise in $m_j(t)$ and the discontinuous transition in $\beta ^{{\rm p}}(t)$.

**Figure 3:**
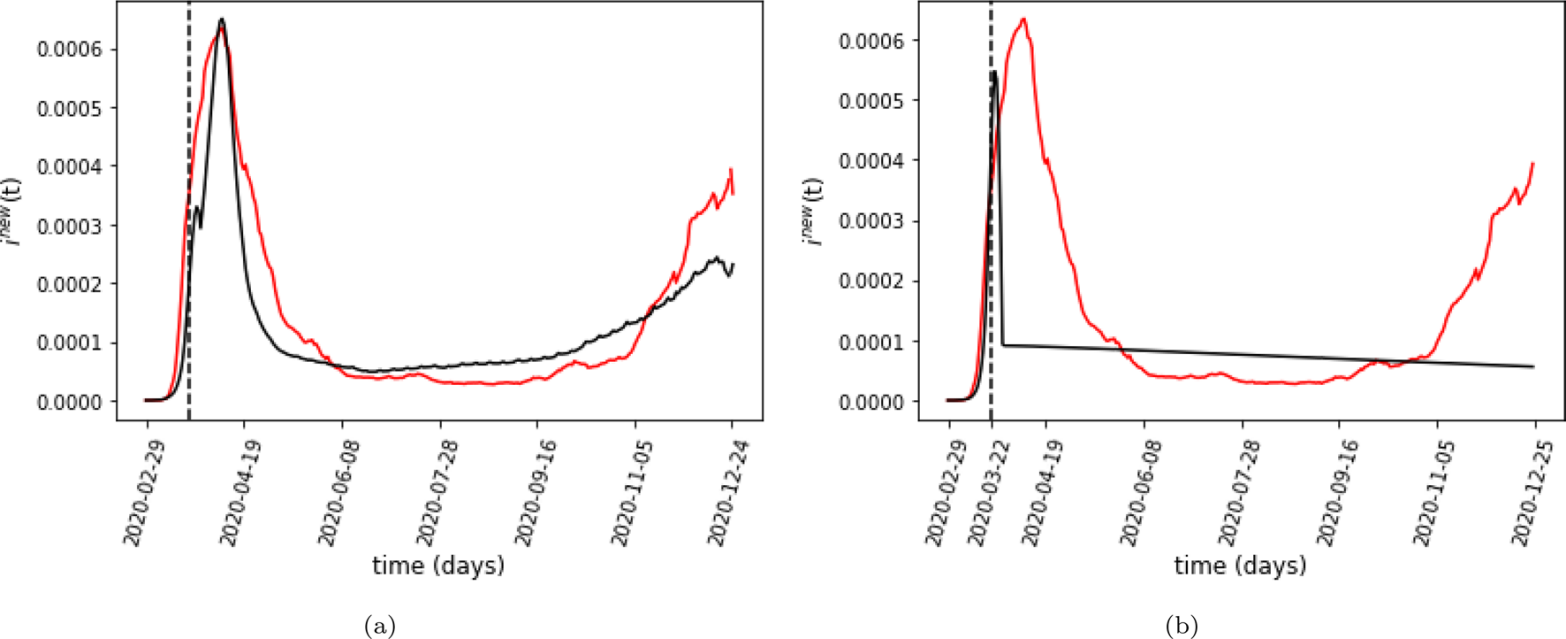
(**a**) The best-fit model output of the daily number of new cases in NYC. The black line shows the model’s output. The red line is the 7-day running average of the total daily reported cases in NYC as a fraction of the total population of the city. The dotted line indicates the start of the NYC Pause Program. (**b**) Fitting results for the model *without* the mobility-dependent infection rate given by Eq. 29. As the plot demonstrates, we cannot fit NYC’s COVID-19 spread without modifying the infection rate by the mobility term.

We have the values of $f_{jj'}(t)$ and ${m_{j}(t)}$ from the data, Using these two values, we can construct $f_{jT}$. We need to learn the values of $\beta ^{ \text {h}}, \beta ^{ \text {l}}, \gamma$ and $\tau _D$. We also need to learn the values of $\beta ^{ \text {h}}_T$ and $\beta ^{ \text {l}}_T$, the infection rate on the subway before and after the start of the PAUSE program. Figure 3a plots the results of fitting the model by minimizing the mean squared error (MSE). We fit our model by sweeping over a million values for these parameters, with our search guided by existing literature on the infection and the recovery rates^28^. By contrast, we can see from Fig. 3b that an SIR model that does not take into account mobility cannot explain the infection trend.

### Forecasting

**Figure 4:**
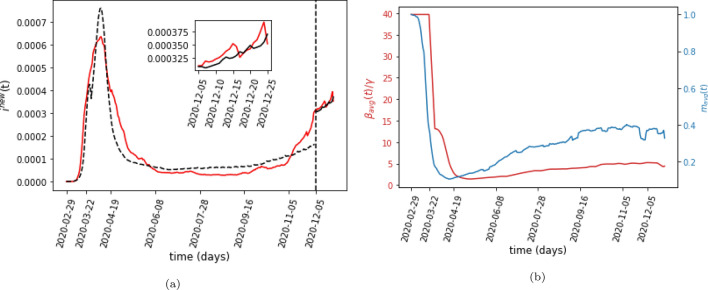
(**a**) The predicted number of daily cases in NYC normalized by the total population of the city. The red line is the 7-day running average of the total daily reported cases in NYC as a fraction of the total population of the city. The dashed black line shows the best-fit output of the model in the training period, and the solid black line shows the model’s prediction for the testing period. The vertical dotted line marks the beginning of the three-week testing period. The inset figure shows the testing period in more detail. (**b**) The ratio of the average infection rate, $\beta _{{\rm avg}}(t) = \sum _j p_j \beta _j(t)$, over the recovery rate, $\gamma$ as a function of time. The second axis shows the average mobility parameter, $m_{{\rm avg}}(t) = \sum _j p_j m_j(t)$.

We also masked the last three weeks of data and trained our model without this period. First, we do a parameter sweep to find the values of the parameters that best fit the training data. Next, we use the end of the training period, $i^{{\rm new}}_{{\rm data}}(t_{{\rm train}})$ (where $t_{{\rm train}}$, is the last day of the training data) as the initial condition for the testing period. However, we cannot directly use the number of daily new cases as the initial condition. Instead, the model requires knowledge of the active infected and total recovered cases, $i(t_{{\rm train}})$ and $r(t_{{\rm train}})$, at the end of the training period as the initial conditions for the testing period. This was not a concern when we were fitting our model for the training period since we assumed that $i(0) = 1/N$ and $r(0) = 0$. In order to predict the spread of the disease in the testing period, however, we need to know these quantities to serve as the initial conditions for our model. While data are available for the number of active and recovered cases for New York state, they are not available for New York City. We estimate the total recovered population by dividing the cumulative deaths reported in New York City by the state-wide case mortality rate^29^

30 $$\begin{aligned} {r_{{\rm est}} (t)}&{=\dfrac{\text {Cumulative deaths reported in NYC on day }t}{N * \text {Case mortality rate on day } t},} \end{aligned}$$

where $r_{{\rm est}} (t)$ is the estimated total recovered population (which includes both individuals who have died as well as those who have recovered from the disease) expressed as a fraction of the total population of New York City. We also need to know $i_{{\rm est}} (t)$, the estimated total active number of infected cases. Specifically, we only need to estimate $i_{{\rm est}} (t_{{\rm train}})$, the total number of active cases on the last day of the training data. We do this by searching for a value of $i_{{\rm est}} (t_{{\rm train}} - 1)$ such that using $i_{{\rm est}} (t_{{\rm train}} - 1)$ and $r_{{\rm est}} (t_{{\rm train}} - 1)$ as the initial condition for our model and predicting the daily number of cases for the next day gives us

31 $$\begin{aligned} {i^{{\rm new}}(t_{{\rm train}})}&{= i^{{\rm new}}_{{\rm data}}(t_{{\rm train}}),} \end{aligned}$$

where $i^{{\rm new}}(t_{{\rm train}})$ are the daily number of new cases output by our model on the last day of the training data. The corresponding values of $i_{{\rm est}} (t_{{\rm train}})$ and $r_{{\rm est}} (t_{{\rm train}})$ become the initial conditions for the model at the start of the testing period. The model’s prediction is shown in Fig. 4a. Table 2 shows the parameters that minimize the MSE with and without a testing period.

**Table 2:** The results from fitting the data with and without the last three weeks masked. $E_{{\rm in}}$ refers to the MSE of fitting the data, while $E_{{\rm out}}$ shows the MSE of the model’s prediction during the testing period. While we minimize the MSE during the fitting process, the table also reports the in-sample and out of sample $R^2$ score of the best fit. We use $\Delta t = 10^{-2}$ for all our simulations.

Parameter values	Data fitting without testing period	Data fitting with three-week testing period
$\beta ^{{\rm h}}$	1.55	1.59
$\beta ^{{\rm l}}$	0.55	0.53
$\beta ^{{\rm h}}_T$	4	4
$\beta ^{{\rm l}}_T$	4	4
$\gamma$	0.04	0.04
${t_D}$	21 days	21 days
$E_{{\rm in}}$	$4.46\times 10^{-9}$	$2.98\times 10^{-9}$
$E_{{\rm out}}$	–	$2.44\times 10^{-10}$
$R^2_{{\rm in}}$	0.82	0.88
$R^2_{{\rm out}}$	–	0.43

## Discussion

Figure 1a shows that the total number of cases in NYC rapidly increased after the discovery of the first recorded case, followed by a decline and then a second rise. This trend seems to follow the usage of the subway: initially, the usage of the subway declines precipitously and then it slowly and partially recovers to about 2/3 of the previous usage.

By scaling our infection parameter with subway usage, we are simultaneously capturing two effects. The first is the rise in infections directly due to the use of the subway, either through higher infection rate or through case importation between regions. The second is taking subway usage as a proxy for broader mobility trends, which in turn depend upon public policy that governs the infection rate. As more people went back to work and as restrictions on public gatherings, schools etc. were eased, we assume that there was a corresponding increase in human mobility proportional to the increase in subway usage, even though this usage is just one form of the total population mobility in the city.

The fact that our model is able to accurately capture both the first wave of infections as well as the second one indicates that our assumption that subway usage is an indicator for broader human mobility trends (and for public policies regarding restrictions more generally) within the city is correct. While our model does predict a higher infection rate for the subway than for the boroughs, infection trends are much less sensitive to inter-region mobility compared to intra-region mobility.

If we set $\beta _j(t) = \beta ^{{\rm p}}(t)$ and the dependence on ${m_j(t)}$ is removed, the reduced model is unable to capture the second wave of infections towards the end of the year as shown in Fig. 3b.

### Limitations

The turnstile data that we use imposes some limitations on our model. The most crucial assumption in our work is that subway usage is correlated to all mobility within the city and can therefore be used as a proxy for all mobility. This assumption is supported by the fact that the usage of both bikeshares and taxis dropped at the same time as that of the subway^19, 20^, and bikeshare usage increased in the same period as subway usage, although at a much faster rate^18^. Additional data on other forms of mobility, specially in the latter half of 2020, would allow us to construct the mobility parameter that encapsulates multiple modes of transport.

We also assume that the residents of a borough that leave it using the subway return to the home borough using the subway on the same day. This assumption impacts our inter-region mixing parameter through the calculation $\Delta \tau _j$, the average time spent away from the home borough.

Finally, we assume that the fraction of cases that were reported remained constant throughout 2020. While we have adjusted for the drop in reporting on the weekends by taking a 7-day moving average, the fraction of cases that were reported may have changed over the course of the year due to other factors as well. A possible effect of this variation in the reporting rate is the very high ratio of the average infection rate, defined as $\beta _{{\rm avg}}(t) = \sum _j p_j \beta _j(t)$, to the recovery rate, $\gamma$, that our model predicts during the beginning of the infection, shown in Fig. 4b. The precipitous rise in cases at the start of the pandemic may represent a slew of people getting tested in a short amount of time as awareness of the epidemic spread and widespread testing became available, rather than accurately representing the true spread of the disease. After this initial period our $\beta _{{\rm avg}}(t)/\gamma$ ratio has a minimum of 1.43 and a maximum of 5.25. While an initial estimate for the reproduction number was reported to be 2.2 in Wuhan^30^, other studies using SIR models have reported a much higher reproduction number ranging from a global estimate of 4.5^31^ to some regions having a value as high as 7.8^32^. While the ratio $\beta _{{\rm avg}}(t)/\gamma$ is not equivalent to the reproduction number (due to the connectivity of the different compartments in our model) and should be seen only as a crude estimate, it is encouraging that the ratio predicted by our model falls within the range of estimates reported in the literature.

## Conclusion

The main contribution of this paper is an introduction of a mobility-based model of epidemic spread that uses a mobility-dependent infection rate. Based only on fitting the data, our model confirms that subway usage is correlated with the usage of other forms of public transportation because using it as a proxy for the short-range mobility parameter allows us to predict the two peaks in the NYC infection rate in 2020. Using this model and the turnstile data from the NYC subway, we predict the trend of daily infections in NYC for a three-week period. Our model accounts for inter-region mixing of populations, and uses an infection rate that is dependent on the short-range mobility within a region.

While we have used NYC as a test case, it would be interesting to verify the model with data from other cities. We believe that by incorporating data from other public transportation services, such as taxis, ride- and bike-sharing services etc., our model can offer more accurate predictions about the spread of an epidemic disease. Thus, it can be a useful tool in guiding public policies to tame the spread of pandemics.

## References

[1] Kraemer MUG, Yang CH, Gutierrez B, Wu CH, Klein B, Pigott DM, du Plessis L, Faria NR, Li R, Hanage WP, Brownstein JS, Layan M, Vespignani A, Tian H, Dye C, Pybus OG, Scarpino SV (2020). The effect of human mobility and control measures on the COVID-19 epidemic in China. Science.

[2] Herrera-Valdez MA, Cruz-Aponte M, Castillo-Chavez C (2011). Multiple outbreaks for the same pandemic: Local transportation and social distancing explain the different “waves” of A-H1N1pdm cases observed in México during 2009. Math. Biosci. Eng..

[3] Troko J, Myles P, Gibson J, Hashim A, Enstone J, Kingdon S, Packham C, Amin S, Hayward A, Van-Tam JN (2011). Is public transport a risk factor for acute respiratory infection?. BMC Infect. Dis..

[4] Harris, J. E. *The subways seeded the massive coronavirus epidemic in New York City*. Working Paper 27021, National Bureau of Economic Research (April 2020).

[5] Fathi-Kazerooni S, Rojas-Cessa R, Dong Z, Umpaichitra V (2021). Correlation of subway turnstile entries and COVID-19 incidence and deaths in New York City. Infect. Dis. Model..

[6] Liu W, Wang D, Hua S, Xie C, Wang B, Qiu W, Tao X, Ye Z, Linling Yu, Yang M, Xiao Y, Feng X, Shi T, Li M, Chen W (2021). Spatiotemporal analysis of Covid-19 outbreaks in Wuhan, China. Sci. Rep..

[7] Tian H, Liu Y, Li Y, Wu C-H, Chen B, Kraemer MUG, Li B, Cai J, Xu B, Yang Q, Wang B, Yang P, Cui Y, Song Y, Zheng P, Wang Q, Bjornstad ON, Yang R, Grenfell BT, Pybus OG, Dye C (2020). An investigation of transmission control measures during the first 50 days of the COVID-19 epidemic in china. Science.

[8] Askitas N, Tatsiramos K, Verheyden B (2021). Estimating worldwide effects of non-pharmaceutical interventions on Covid-19 incidence and population mobility patterns using a multiple-event study. Sci. Rep..

[9] New York State Department of Health. New York State on PAUSE. https://coronavirus.health.ny.gov/new-york-state-pause.

[10] Reichert TA, Sugaya N, Fedson DS, Glezen WP, Simonsen L, Tashiro M (2001). The Japanese experience with vaccinating schoolchildren against influenza. N. Engl. J. Med..

[11] Carrión D, Colicino E, Pedretti NF, Arfer KB, Rush J, DeFelice N, Just AC (2021). Neighborhood-level disparities and subway utilization during the Covid-19 pandemic in New York City. Nat. Commun..

[12] Kissler SM, Kishore N, Prabhu M, Goffman D, Beilin Y, Landau R, Gyamfi-Bannerman C, Bateman BT, Snyder J, Razavi AS, Katz D, Gal J, Bianco A, Stone J, Larremore D, Buckee CO, Grad YH (2020). Reductions in commuting mobility correlate with geographic differences in sars-cov-2 prevalence in New York City. Nat. Commun..

[13] Verma R, Yabe T, Ukkusuri SV (2021). Spatiotemporal contact density explains the disparity of Covid-19 spread in urban neighborhoods. Sci. Rep..

[14] Shi, Y., & Ban, X. Capping mobility to control Covid-19: A collision-based infectious disease transmission model. *medRxiv* (2020).

[15] Meloni S, Perra N, Arenas A, Gómez S, Moreno Y, Vespignani A (2011). Modeling human mobility responses to the large-scale spreading of infectious diseases. Sci. Rep..

[16] Metropolitan Transportation Authority. Turnstile data. http://web.mta.info/developers/turnstile.html.

[17] New York Governor’s Office. At novel coronavirus briefing, governor cuomo declares state of emergency to contain spread of virus. https://www.governor.ny.gov/news/novel-coronavirus-briefing-governor-cuomo-declares-state-emergency-contain-spread-virus.

[18] Wang H, Noland RB (2021). Bikeshare and subway ridership changes during the Covid-19 pandemic in New York City. Transp. Policy.

[19] Teixeira JF, Lopes M (2020). The link between bike sharing and subway use during the COVID-19 pandemic: The case-study of New York’s Citi bike. Transp. Res. Interdiscip. Perspect..

[20] Manley E, Ross S, Zhuang M (2021). Changing demand for New York yellow cabs during the Covid-19 pandemic. Findings.

[21] RSG. Metropolitan Transportation Authority New York City Travel Survey. Technical report, 06 2020.

[22] New York City Department of Health and Mental Hygiene. Covid-19: Data. https://www1.nyc.gov/site/doh/covid/covid-19-data.page.

[23] United States Census Bureau. 2020 census. https://data.census.gov/cedsci/ (2020).

[24] Kermack WO, McKendrick AG (1991). Contributions to the mathematical theory of epidemics-i. Bull. Math. Biol..

[25] Newman MEJ (2010). Networks: An Introduction.

[26] Mwalili S, Kimathi M, Ojiambo V, Gathungu D, Mbogo R (2020). Seir model for Covid-19 dynamics incorporating the environment and social distancing. BMC. Res. Notes.

[27] Wielechowski M, Czech K, Grzeda Ł (2020). Decline in mobility: Public transport in Poland in the time of the COVID-19 pandemic. Economies.

[28] Johansson MA, Quandelacy TM, Kada S, Prasad PV, Steele M, Brooks JT, Slayton RB, Biggerstaff M, Butler JC (2021). SARS-CoV-2 transmission from people without COVID-19 symptoms. JAMA Netw. Open.

[29] Johns Hopkins University. Covid-19 data repository by the center for systems science and engineering (csse) at Johns Hopkins University. https://github.com/CSSEGISandData/COVID-19 (2021).

[30] Li Q, Guan X, Wu P, Wang X, Zhou L, Tong Y, Ren R, Leung KSM, Lau EHY, Wong JY, Xing X, Xiang N, Wu Y, Li C, Chen Q, Li D, Liu T, Zhao J, Liu M, Tu W, Chen C, Jin L, Yang R, Wang Q, Zhou S, Wang R, Liu H, Luo Y, Liu Y, Shao G, Li H, Tao Z, Yang Y, Deng Z, Liu B, Ma Z, Zhang Y, Shi G, Lam TTY, Wu JT, Gao GF, Cowling BJ, Yang B, Leung GM, Feng Z (2020). Early transmission dynamics in Wuhan, china, of novel coronavirus-infected pneumonia. N. Engl. J. Med..

[31] Katul GG, Mrad A, Bonetti S, Manoli G, Parolari AJ (2020). Global convergence of Covid-19 basic reproduction number and estimation from early-time sir dynamics. PLoS ONE.

[32] You C, Deng Y, Hu W, Sun J, Lin Q, Zhou F, Pang CH, Zhang Y, Chen Z, Zhou X-H (2020). Estimation of the time-varying reproduction number of Covid-19 outbreak in china. Int. J. Hyg. Environ. Health.

